# Respiratory syncytial virus-associated pneumonia in primary care in Malawi

**DOI:** 10.1093/tropej/fmae013

**Published:** 2024-07-18

**Authors:** Kimberly Davy, Elena Koskinas, Chris Watson, Mark Ledwidge, Balwani Mbakaya, Master Chisale, Joe Gallagher

**Affiliations:** School of Medicine, University of Limerick, Limerick V94 T9PX, Ireland; School of Medicine, University of Limerick, Limerick V94 T9PX, Ireland; Medicine, Health and Life Sciences, Queen’s University Belfast Wellcome-Wolfson Institute for Experimental Medicine, Belfast BT9 7BL, UK; School of Medicine, University College Dublin, Dublin D04 C1P1, Ireland; Department of Public Health, University of Livingstonia, Mzuzu P.O. 112, Malawi; Biological Science Department, Faculty of Science Technology and Innovations, Mzuzu University, Mzuzu, P / Bag 20, Malawi; Department of General Practice, University College Dublin, University College Dublin, Dublin, Belfield, Dublin 4, D04 C1P1, Ireland

**Keywords:** respiratory syncytial virus, primary care, influenza, paediatrics, RSV vaccination, Malawi

## Abstract

**Objective:**

To identify the prevalence of respiratory syncytial virus (RSV) in a cohort of children under 5 years of age with World Health Organization (WHO)-defined pneumonia and the factors associated with developing severe RSV-associated community-acquired pneumonia (CAP) in primary care in a single centre in Northern Malawi.

**Methods:**

The BIOmarkers TO diagnose PnEumonia (BIOTOPE) study was a prospective cohort study conducted from March to June 2016 that took place in a primary care centre in Northern Malawi. Data from this study was used to identify the characteristics of children under 5 years of age who presented with RSV and WHO-defined CAP. Means, standard deviations, medians and ranges were calculated for continuous variables. A univariate logistic regression was performed to examine the potential predictor variables.

**Results:**

Four hundred and ninety-four infants presented with CAP and were eligible for inclusion in the study; RSV infection was detected in 205 (41.6%) of the infants. Eight factors were associated with increased risk for RSV CAP in the univariate model: age, born at term, presenting for care in June, crowded living environment, not being exclusively breastfed, not having received zinc or vitamin A supplementation in the last six months. Infants with RSV were more likely to have an oxygen saturation ≤92% compared to infants with other causes of pneumonia and more likely to have severe pneumonia as defined by the WHO.

**Conclusion:**

This study supports that RSV-associated CAP is linked to modifiable and non-modifiable risk factors; further research is indicated to determine which interventions would be most impactful. Developing and implementing an infant or maternal vaccine could be a cost-effective way to prevent RSV-associated CAP and mortality in developing nations. More research is needed to understand seasonal patterns of CAP and research over extended periods can offer valuable insights on host, environmental and pathogen-specific factors that contribute to RSV-associated CAP.

## INTRODUCTION

Community-acquired pneumonia (CAP) causes substantial morbidity and mortality in children under five years of age globally, with a disproportionately large number of cases in sub-Saharan Africa [1]. Respiratory viruses, particularly respiratory syncytial virus (RSV), significantly contribute to CAP and childhood mortality, as evidenced by the three million hospitalizations and 60 000 annual deaths worldwide [2]. Children under six months assume half of these hospitalizations and deaths [2]. In Malawi 11.9% of children with severe acute respiratory infection had RSV and this had an adjusted risk ratio of 1.9 for severe infection [3]. The mean cost per RSV episode in Malawi is $62.26 USD per inpatient case, which is approximately one-third of the average monthly household income [4]. Contemporary estimates of the microbiological causes of pneumonia and predictors of hospitalization from primary care settings would be of benefit since it is in the community where these patients are most likely to present [5]. In particular, the analysis of the burden of RSV in the community would help determine the role of prevention strategies such as immunization [5].

Decades of surveillance data on RSV in high-income nations have demonstrated that RSV causes yearly seasonal outbreaks. The timing and duration of the outbreaks vary geographically and can be predicted by location from year to year. However, pathogen-specific data is scarce or non-existent in low-income nations. The surveillance data available on RSV is limited to hospitalized patients and incomplete due to differences in reporting and a lack of diagnostic resources available. The landmark Pneumonia Etiology Research for Child Health (PERCH) study provided insight into variables associated with developing severe RSV; however, the participants were solely hospitalized patients [6]. Studies investigating the risk factors associated with increased RSV disease severity in primary care are lacking in Africa. An analysis of different host factors like age, gender, premature birth and nutritional status; environmental factors such as higher house population density and increased smoke exposure; and pathogen-specific factors that affect children with RSV would be important to help address the associated morbidity and mortality [5].

This aim of this paper is to determine the prevalence and risk factors for RSV in the primary care cohort in the BIOTOPE study (BIOmarkers TO diagnose PnEumonia)—a prospective cohort study that systematically enrolled children presenting with CAP to primary care in Northern Malawi [5, 7].

## METHODS

The study describes the characteristics of children under 5 years of age with RSV and WHO-defined pneumonia from the BIOmarkers TO diagnose PnEumonia (BIOTOPE) study [5]. In brief, participants were patients that presented to the outpatient department of Mzuzu Central Hospital and Mapale Health Centre (a primary care centre) with WHO-defined pneumonia from March to June 2016. Both sites serve as primary care facilities for the urban area of Mzuzu in Northern Malawi. Further details on the site and methods are available in an online Supplementary file (https://bmjopen.bmj.com/content/11/7/e046633#DC1). The study protocol and related documents were approved by the National Health Science Research Committee of Malawi and the Mzuzu Central Hospital Research Committee. National Health Research Committee of Malawi Ethics Approval #15/11/1532.

The final diagnosis was determined based on pre-determined clinical and microbiological assessment criteria (Supplementary file).

### Statistical analysis

The overall aim of this study was to determine the prevalence and risk factors that contribute to children presenting with RSV-associated CAP. Categorical variables were expressed as percentages. Means, standard deviations, medians and ranges were calculated for continuous variables. A univariate logistic regression was performed to examine the potential predictor variables. Low birthweight included participants <2.5 kg, household crowding consisted of ≥3 people sleeping in the same room as the participant, and secondary school complete was defined as 12 years of education completed. Exclusive breastfeeding was indicated as infants exclusively breastfed until six months of age. A *p*-value of ≤0.05 was considered statistically significant. The data was fitted to a stepwise logistic regression model to determine variables significantly associated with a positive RSV finding. Odds ratios and 95% confidence intervals were calculated. All analyses were performed using Microsoft Excel for Mac version 16.84 (Microsoft Corporation, Microsoft Excel for Mac version 16.84, Seattle, WA, USA).

## RESULTS

The study group included 494 infants with CAP, of which RSV infection was detected in 205 (41.6%) children. There were 86 (42.0%) infants who were under 12 months old in the RSV-positive group compared to 71 (24.6%) in the RSV-negative group (*p* < 0.001). Of study participants with health passports, 374 (94%) had the pneumococcal conjugate vaccine (PCV13). The sociodemographic, environmental and clinical characteristics of the two groups are presented in Tables 1 and 2. Children positive for RSV-associated CAP were more likely to be younger, born at term, presented in June, lived in crowded spaces, had not been exclusively breastfed, had not received zinc or vitamin A supplementation in the last six months in a univariate analysis. Children with RSV were also more likely to have had an oxygen saturation ≤92% and severe pneumonia (as defined by WHO) in a univariate analysis.

**Table 1.** Demographic data.

Demographics	Total population in study	RSV (+) CAP^a^	RSV (−) CAP	Odds Ratio (OR)	*p*-value
	*N* = 494	*N* = 205	*N* = 289	(95% CI)	
Mean age in months (SD)		1.46 (1.2)	1.96 (1.47)		
2 to <12 months	157/494 (31.8%)	86/205 (42.0%)	71/289 (24.6%)	2.21 (1.51 to 3.26)	<0.001
12 to < 36 months	252/494 (51.0%)	100/205 (48.8%)	152/289 (52.6%)	0.86 (0.6 to 1.23)	0.70
>36 months	85/494 (17.2%)	19/205 (9.3%)	66/289 (22.8%)	0.35 (0.20 to 0.60)	<0.001
Sex (male)	271/494 (54.9%)	106/204 (51.7%)	165/289 (57%)	0.81 (0.57 to 1.17)	0.26
March	12/492 (12.4%)	0/205 (0%)	12/289 (4.2%)	0.05 (0.003 to 0.92)	0.04
April	40/492 (8.1%)	3/205 (1.5%)	37/289 (13.8%)	0.10 (0.03 to 0.33)	0.0002
May	184/492 (37.4%)	34/205 (16.6%)	150/289 (51.9%)	0.18 (0.12 to 0.29)	<0.0001
June	256/492 (52%)	168/205 (82.0%)	88/289 (30.4%)	10.37 (6.71 to 16.03)	<0.0001
Birth weight <2.5 kg	31/462 (6.7%)	10/200 (5%)	21/262 (8%)	0.60 (0.28 to 1.31)	0.20
Prematurity <37 weeks	35/485 (7.2%)	8/204 (3.9%)	27/281 (9.6%)	0.38 (0.17 to 0.86)	0.02
Cigarette smoke exposure	42/494 (8.5%)	14/205 (6.83.%)	28/289 (9.69%)	0.399 (0.21 to 0.74)	0.0039
Household crowding	433/494 (87.7%)	187/205 (91.2%)	246/289 (85%)	1.8 (1.01 to 3.25)	0.045
Exclusively breastfed	329/491 (67%)	121/205 (59%)	208/286 (72.7%)	0.54 (0.37 to 0.79)	0.0015
Maternal education	86/494 (17.4%)	39/205 (19%)	47/289 (16.3%)	1.21 (0.76 to 1.93)	0.426
Zinc or vitamin A supplementation	79/494 (16.0%)	19/205 (9.3%)	60/289 (20.8%)	0.39 (0.22 to 0.68)	0.0008
Charcoal used as primary fuel source	299/494 (60.5%)	138/205 (67.3%)	161/289 (55.7%)	1.21 (0.91 to 1.61)	0.20

a RSV - Respiratory Syncytial Virus; CAP - Community acquired pneumonia; OR - Odds ratio

**Table 2.** Clinical features and severity

	Total population in study	RSV (+) CAP^a^	RSV (−) CAP	Adjusted OR (95% CI)	*p*-value
	N = 494	*N* = 205	*N* = 289		
Temperature	*N* = 471	*N* = 197	*N* = 275	0.84	0.378
Mean (SD)	37.5 (1.03)	37.5 (0.96)	37.6 (1.08)	(0.58 to 1.23)	
Respiratory rate	59.6 (9.5)	60 (8.5)	59.5 (10.2)	1.34 (0.94 to 1.92)	0.111
Oxygen saturation <92%	80 (16.2%)	42 (20.5%)	38 (13.1%)	1.70 (1.05 to 2.75)	0.03
Severe pneumonia (as defined by WHO Intergrated Management of Childhood Illness (IMCI) criteria on hospitalized patients)	62 (12.6%)	39 (19.0%)	23 (8.0%)	2.72 (1.57 to 4.71)	0.0004
Hospitalizations	56 (11.3%)	26 (12.7%)	30 (10.4%)	1.25 (0.72 to 2.20)	0.427

a RSV - Respiratory Syncytial Virus; CAP - Community acquired pneumonia; OR - Odds ratio

**Figure 1:**
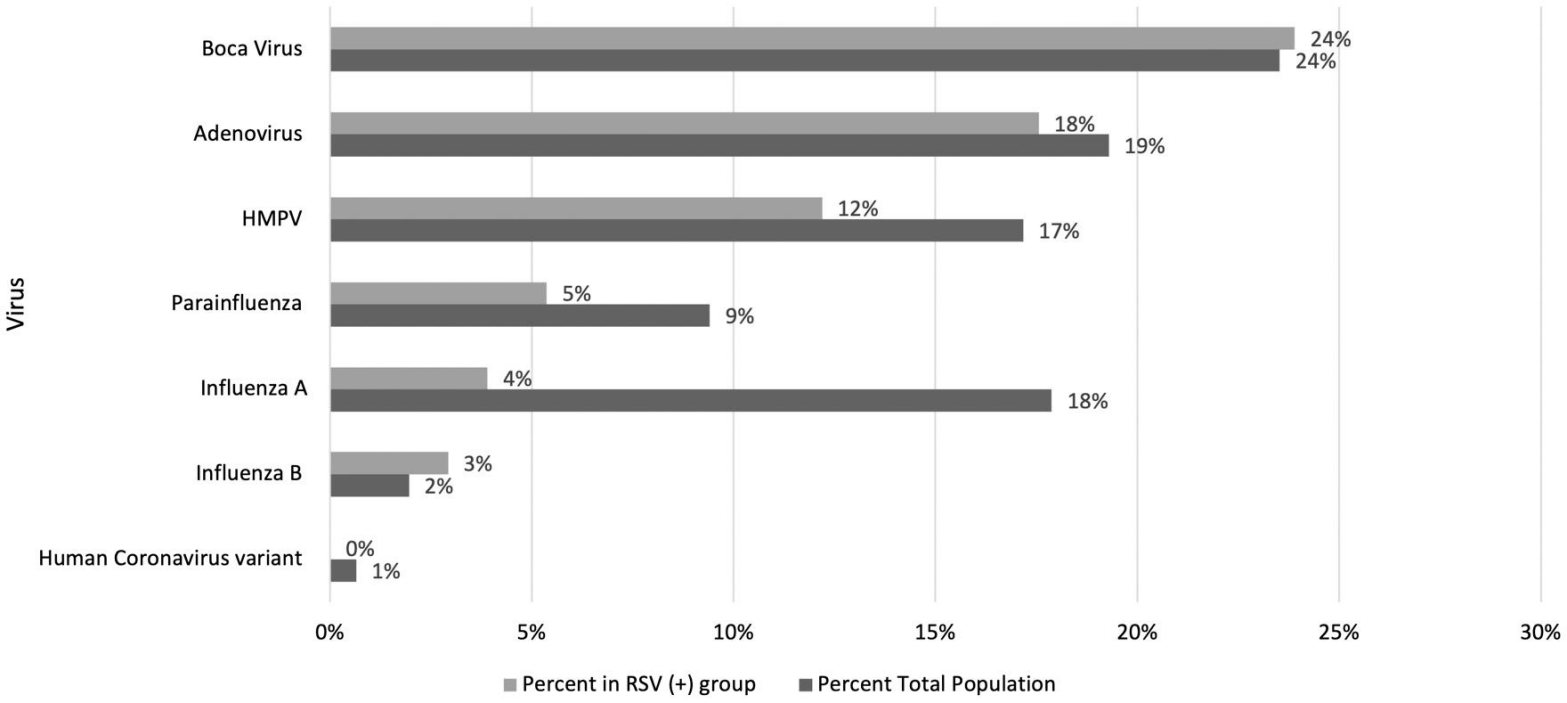
Distribution of other viral pathogens in children with RSV-associated pneumonia, compared to the total population studied.

Figure 1 shows the distribution of co-detected viral pathogens in children with RSV pneumonia. Bocavirus was the commonest virus detected in RSV CAP (24%). The percentage of RSV-negative CAP concurrently infected with Bocavirus was lower at 18%. Bacteria were only found in 4.9% of participants with RSV CAP.

### Predictors of RSV CAP

Multivariate regression was performed to examine the predicators of RSV-associated CAP. All variables with *p* < 0.05 in the univariate analysis were considered for inclusion. The month of infection, age and non-exclusive breastfeeding were most associated with RSV CAP in the univariate analysis. In June, pneumonia in a child is 1.29 times more likely to be due to RSV than other viral or bacterial pathogens. The odds of RSV-associated CAP were 1% higher in a participant who was not breastfed exclusively. Nutritional supplementation (with zinc or vitamin A) also decreased the odds of RSV-associated CAP by 7.6%. Table 3 outlines notable predictors of RSV-associated CAP.

**Table 3.** Predictors of RSV (+) pneumonia

	Regression coefficient B	Exp (B)	95% CI for Exp (B)		*p*-value
			Lower	Upper	
Month	0.256	1.29	0.203	0.310	<0.001
Age	−0.057	0.945	−0.091	−0.023	<0.001
Exclusively breastfed	−0.008	0.992	−0.083	0.067	0.841
Premature	−0.115	0.891	−0.267	0.037	0.138
Nutritional supplementation	−0.080	0.924	−0.189	0.031	0.160
Oxygen saturation	0.070	1.072	−0.038	0.176	0.204

*R* ^2^ = 0.226 or 22.6% (coefficient of determination). *p* < 0.001. Exp - exponential; CI - Confidence interval.

## DISCUSSION

This study examined the prevalence and predictors of RSV-associated CAP among children presenting to primary care in Malawi. RSV was one of the most commonly detected viruses in children, which matched previous studies from low-income countries [8, 9]. The variables most associated with contracting RSV were found to be multifactorial. Demographic factors include age 2–12 months and born at term in a univariate analysis. Age greater than 36 months was associated with reduced probability of having RSV-associated CAP. Environmental factors include month of infection and more than three people sleeping in the same room. Other factors include lack of exclusive breastfeeding, no zinc or vitamin A supplementation in the last six months. Children with RSV pneumonia were more likely, than children with pneumonia due to other pathogens to have an oxygen saturation ≤92% and severe pneumonia as defined by the WHO [7]. The final regression model showed that age and month of infection were significant predictors of RSV-positive CAP when controlling for covariates.

Household crowding has been consistently associated with an increased risk of acquiring RSV-associated infection regardless of the definition of household crowding [10]. RSV spreads through viral shedding under close conditions; those in close contact with RSV-infected individuals are at risk of exposure to the virus during maximal shedding timing (early in infection) and receive higher amounts of the inoculate [10]. Educational interventions which target infection prevention control measures (mask-wearing, hand washing) could decrease the risk of RSV infections in overcrowded households.

Non-breastfeeding practices have been similarly cited in the literature as posing significant risk for RSV-associated lower respiratory tract infection (LRTIs) [11, 12]. Mineva, *et al.* [11] identified that exclusive breastfeeding for >4–6 months significantly lowered hospitalization, length of stay, supplemental oxygen demand and admission to intensive care units. Education on breastfeeding principles in developing countries could serve as effective primary prevention strategies. Factors contributing to exclusive breastfeeding include the mother’s age, ethnicity of the mother, sex of the infant, number of children of the mother and decision-makers within the family. Previous studies have suggested targeted interventions to promote exclusive breastfeeding that includes community breastfeeding groups, health education and awareness campaigns, the use of mass media, and the re-examination of the Baby-Friendly Hospital Initiative and Baby-Friendly Community Initiative [13, 14].) 

There is an increased focus on RSV given its impact on child health and health services. Until recently the only medical prevention strategy available was palivizumab, a monoclonal antibody indicated in a specific subset of infants with comorbidities or delivered preterm and more recently nirsevimab is a monoclonal antibody to the RSV fusion protein with an extended half-life that could provide promising outcomes [15].

### Prevention of RSV in infants

There is an increased focus on RSV given its impact on child health and health services. Palivizumab (Synagis TM) is the current standard of preventative care licenced for infants at the highest risk for severe RSV disease. Although it is expensive and intensive to administer which is a challenge for low-resource countries, it decreases hospitalization due to RSV [23]. This includes those born preterm (GA < 35 weeks), younger than six months at the start of the RSV season, and children younger than two years of age with chronic lung disease of prematurity or hemodynamically significant congenital heart disease [24]. Prophylaxis is started at the beginning of the RSV season and given via intramuscular injection monthly throughout the season [25]. The approximate cost of one milligram of palivizumab was 8.25 Euro, equivalent to approximately 15 113 Malawian Kwacha, in 2020 [26]. A one-time vaccine may be a less costly and burdensome alternative, especially in developing nations where access to primary care is limited.

More recently, many developed nations worldwide including the European Union, the UK, Canada and the USA have authorized the use of Nirsevimab (Beyfortus TM), a long-acting monoclonal antibody to the RSV fusion protein that offers 5 months of protection for RSV in infants [24]. A single intramuscular injection before a late-term or term infant’s first RSV season demonstrated efficacy in protecting against medically attended RSV infections. As a result, it is recommended that all infants less than 8 months of age during their initial RSV season receive a single dose of nirsevimab [27]. Additionally, children with chronic lung disease of prematurity requiring medical support in the 6 months before their second RSV season, those with severe immunocompromise, those with congenital heart disease, or children with cystic fibrosis are advised to receive nirsevimab upon entering their second RSV season [24]. These recommendations could change and expand as more research specifically targets low-and middle-income countries. Nirsevimab necessitates only one payment and one visit to a primary care centre in a single season. Its affordability in low-and middle-income countries remains uncertain [24, 28].

### Maternal vaccination

Worldwide, phase 3 trials, have shown preliminary evidence of safety and efficacy of preventing medically attended severe RSV-associated LRTI in infants following maternal vaccination with a bivalent RSV prefusion F protein-based (RSVpreF) vaccine at 24–36 weeks of pregnancy [28, 29]. Immunizing mothers with RSVpreF vaccines was found to produce neutralizing antibody responses approximately seven weeks after immunization and could adequately transfer transplacentally [29].

The MATISSE study [28] on RSVpreF vaccine involved patients from Gambia and South Africa (15.7% of total study population).

The evidence is promising thus far, but future studies must address vaccine efficacy in the long term and the logistical approach for its use in sub-Saharan Africa primary care settings. As the research on vaccinating healthy pregnant mothers continues, so should consideration about implementing these as preventative measures in the primary care setting of sub-Saharan African countries. As can be seen in this study there is high uptake of other vaccines in the current programme which highlights the potential to introduce these vaccines into the Malawian vaccination schedule [28].

Presently, RSV vaccinations are available in specific populations within high-income countries. For adults aged 60 or older, Arexvy is administered as a two-vaccine series. Additionally, pregnant women can receive Abrysvo as an RSV vaccine during weeks 32 through 36 of pregnancy [30]. An RSV vaccine has not yet been approved for use in children [30,31]. A recent systematic review highlighted the cost and requirement of access to a dose every month mean that palivizumab is a less appealing option in low-income and middle-income countries and highlighted that the uncertainty as to whether these interventions will be cost-effective in low- and middle-income settings at current prices. Further work has highlighted that this does depend on cost. For maternal vaccination, a dose price of 40 USD may be cost-effective for Kenya and South Africa and a dose price of 10 USD cost saving in South Africa [32,33]. A table summarizing these strategies is available in the Supplementary file.

Vitamin A deficiency can lead to heightened susceptibility to infections, resulting in increased morbidity and mortality [16]. In Malawi, the prevalence of vitamin A deficiency has decreased due to the introduction of vitamin A-fortified staple foods and biannual high-dose vitamin A supplementation. However, this success has raised concerns about potential vitamin A excess, prompting a re-evaluation of the universal approach to vitamin A supplementation [17]. Similarly, zinc is involved in multiple body processes including immune system performance and DNA replication [18]. Zinc deficiency in early childhood has been linked to increased risk of diarrhoea and pneumonia [18,20]. During the first six months of breastfeeding, infants receive sufficient zinc intake. However, after this period, infants in sub-Saharan Africa predominantly rely on plant-derived food sources, putting them at risk of zinc deficiency. There has been evidence to support the use of zinc supplementation in RSV and other respiratory illnesses in children with one study demonstrating a 21% reduction in pneumonia incidence in children 12–59 months of age [18, 19]. Previous research has demonstrated that exposure to environmental tobacco smoke puts infants and young children at an increased risk of developing severe RSV [21, 22]. Our study did not confirm the finding; however this is likely due to fact that only a small percentage (42/494 or 8.5%) of participants were exposed tobacco smoke; and none of the participants under 12 months of age were exposed to tobacco smoke. Thus interventions to minimize tobacco smoke exposure may not be as valuable in Northern Malawi.

### Limitations

Participants were recruited for our study from March to June 2016. The study was conducted during one season in an urban area with high immunization rates believed to be representative of Malawi and the Mzuzu region [5]; for this reason, we believe our study’s findings may be generalized and applied to Malawi as a nation. The results of this study may also be cautiously generalizable to other countries of sub-Saharan Africa. It has been noted that significant variability in infection rates exists depending on the season; had this study been conducted throughout a different season or for 12 months, the results may have differed [5]. RSV season in Southern Africa has been cited as lasting from October to May, peaking in March [18]. More research is required to adequately compare RSV’s seasonal patterns and other pneumonia causes. Our study found that RSV was most prevalent during June, while other causes of pneumonia were evenly distributed across the four-month study period. It is worth noting, however, that the recent COVID-19 pandemic and its associated restrictions may change the apparent seasonal patterns of RSV [19]. In particular, delayed RSV outbreaks have been observed due a lack of exposure from the previous season resulting in decreased protective immunity within communities [19].

## CONCLUSION

RSV-associated CAP contributes to significant morbidity and mortality in Malawi. RSV is associated with younger age, crowded housing, lack of exclusive breastfeeding, and lack of vitamin A and zinc supplementation. Community interventions that target primary prevention, including proper infection prevention control measures in the household and exclusive breastfeeding practices may help decrease the burden of RSV-associated CAP presenting to primary care. Developing and implementing a vaccine given to the infant or mother could be an effective, less burdensome and more economical intervention to prevent RSV-associated CAP and mortality in Malawi. Further study is warranted to determine the seasonal pattern of RSV-associated CAP compared to other causes of pneumonia which may also have changed following the COVID 19 pandemic. Additional research over extended periods (> 1 year) would be valuable to develop further evidence on host, environmental and pathogen-specific factors contributing to RSV-associated pneumonia.

## Supplementary Material

fmae013_Supplementary_Data

## References

[1] Yan X-L , Li Y-N, Tang Y-J, et al Clinical characteristics and viral load of respiratory syncytial virus and human metapneumovirus in children hospitalised for acute lower respiratory tract infection. J Med Virol 2016;89:589–97. 27632796 10.1002/jmv.24687 PMC7166468

[2] Shi T , McAllister DA, O'Brien KL, et al; RSV Global Epidemiology Network. Global, regional, and national disease burden estimates of acute lower respiratory infections due to respiratory syncytial virus in young children in 2015: a systematic review and modelling study. Lancet 2017;390:946–58. 28689664 10.1016/S0140-6736(17)30938-8 PMC5592248

[3] Peterson I , Bar-Zeev N, Kennedy N, et al Respiratory virus-associated severe acute respiratory illness and viral clustering in Malawian children in a setting with a high prevalence of HIV infection, malaria, and malnutrition. J Infect Dis 2016;214:1700–11. 27630199 10.1093/infdis/jiw426 PMC5341080

[4] Suryadevara M , Domachowske JB. Epidemiology and seasonality of childhood respiratory syncytial virus infections in the tropics. Viruses 2021;13:696. 33923823 10.3390/v13040696 PMC8074094

[5] Gallagher J , Chisale M, Das S, et al; BIOTOPE Team. Aetiology and severity of childhood pneumonia in primary care in Malawi: a cohort study. BMJ Open 2021;11:e046633. 10.1136/bmjopen-2020-046633 PMC8323352 34326047

[6] O'Brien KL , Baggett HC, Brooks WA, et al Causes of severe pneumonia requiring hospital admission in children without HIV infection from Africa and Asia: the PERCH multi-country case-control study. Lancet 2019;394:757–79. 31257127 10.1016/S0140-6736(19)30721-4 PMC6727070

[7] Revised WHO Classification and Treatment of Pneumonia in Children at Health Facilities: Evidence Summaries [Internet]. Geneva: World Health Organization, 2014. https://www.ncbi.nlm.nih.gov/books/NBK264162/ (13 Aug 2023, date last accessed). 25535631

[8] Bénet T , Picot VS, Awasthi S, et al Severity of pneumonia in under 5-year-old children from developing countries: a multicenter, prospective, observational study. Am J Trop Med Hyg 2017;97:68–76. 28719310 10.4269/ajtmh.16-0733 PMC5508893

[9] Divarathne M , Ahamed R, Noordeen F. The impact of RSV-associated respiratory disease on children in Asia. J Pediatr Infect Dis 2018;14:79–88. 32300274 10.1055/s-0038-1637752 PMC7117084

[10] Colosia AD , Masaquel A, Hall CB, et al Residential crowding and severe respiratory syncytial virus disease among infants and young children: a systematic literature review. BMC Infect Dis 2012;12:95. 22520624 10.1186/1471-2334-12-95 PMC3405464

[11] Mineva GM , Purtill H, Dunne CP, et al Impact of breastfeeding on the incidence and severity of respiratory syncytial virus (RSV)-associated acute lower respiratory infections in infants: a systematic review highlighting the global relevance of primary prevention. BMJ Glob Health 2023;8:e009693. 10.1136/bmjgh-2022-009693 PMC9906265 36746518

[12] Pandolfi E , Gesualdo F, Rizzo C, et al Breastfeeding and respiratory infections in the first 6 months of life: a case-control study. Front Pediatr 2019;7:152. 31106183 10.3389/fped.2019.00152 PMC6492465

[13] Salim YM , Stones W. Determinants of exclusive breastfeeding in infants of six months and below in Malawi: a cross sectional study. BMC Pregnancy Childbirth 2020;20:530. 32917175 10.1186/s12884-020-03232-z PMC7488510

[14] Walsh A , Pieterse P, Mishra N, et al Improving breastfeeding support through the implementation of the baby-friendly hospital and community initiatives: a scoping review. Int Breastfeed J 2023;18:22. 37061737 10.1186/s13006-023-00556-2 PMC10105160

[15] “Vitamin A Supplementation in Children 6–59 Months of Age with Severe Acute Malnutrition.” World Health Organization, www.who.int/tools/elena/interventions/vitamina-sam (Accessed 4 Feb 2024, date last accessed).

[16] Fawzi WW , Wang D. When should universal distribution of periodic high-dose vitamin A to children cease? Am J Clin Nutr 2021;113:769–71. 33751042 10.1093/ajcn/nqaa428

[17] Williams AM , Tanumihardjo SA, Rhodes EC, et al Vitamin A deficiency has declined in Malawi, but with evidence of elevated vitamin A in children. Am J Clin Nutr 2021;113:854–64. 33751046 10.1093/ajcn/nqab004 PMC8023849

[18] Brown KH , Hess SY, Vosti SA, et al Comparison of the estimated cost-effectiveness of preventive and therapeutic zinc supplementation strategies for reducing child morbidity and mortality in sub-Saharan Africa. Food Nutr Bull 2013;34:199–214. 23964393 10.1177/156482651303400209

[19] Basnet S , Shrestha PS, Sharma A, et al; Zinc Severe Pneumonia Study Group. A randomized controlled trial of zinc as adjuvant therapy for severe pneumonia in young children. Pediatrics 2012;129:701–8. 22392179 10.1542/peds.2010-3091

[20] Victora CG , Christian P, Vidaletti LP, et al Revisiting maternal and child undernutrition in low-income and middle-income countries: variable progress towards an unfinished agenda. Lancet 2021;397:1388–99. 33691094 10.1016/S0140-6736(21)00394-9 PMC7613170

[21] DiFranza JR , Masaquel A, Barrett AM, et al Systematic literature review assessing tobacco smoke exposure as a risk factor for serious respiratory syncytial virus disease among infants and young children. BMC Pediatr 2012;12:81. 22721493 10.1186/1471-2431-12-81 PMC3411420

[22] Öberg M , Jaakkola MS, Woodward A, et al Worldwide burden of disease from exposure to second-hand smoke: a retrospective analysis of data from 192 countries. Lancet 2011;377:139–46. 21112082 10.1016/S0140-6736(10)61388-8

[23] Garegnani L , Styrmisdóttir L, Rodriguez PR, et al Palivizumab for preventing severe respiratory syncytial virus (RSV) infection in children. Cochrane Database Syst Rev 2021;11:CD013757. 34783356 10.1002/14651858.CD013757.pub2 PMC8594174

[24] Hammitt LL , Dagan R, Yuan Y, et al; MELODY Study Group. Nirsevimab for prevention of RSV in healthy late-preterm and term infants. N Engl J Med 2022;386:837–46. 35235726 10.1056/NEJMoa2110275

[25] Zar HJ , Madhi SA, White DA, et al Acute viral bronchiolitis in South Africa: strategies for management and prevention. S Afr Med J 2016;106:27–9. 27303780

[26] Fernlund E , Eriksson M, Söderholm J, et al Cost-effectiveness of palivizumab in infants with congenital heart disease: a Swedish perspective. J Congenit Heart Dis 2020;4:1–12.

[27] Nirsevimab frequently asked questions. American Academy of Pediatrics. https://www.aap.org/en/patient-care/respiratory-syncytial-virus-rsv-prevention/nirsevimab-frequently-asked-questions/#:∼:text=A%20dose%20of%20nirsevimab%20is,is%20inclusive%20of%2019%20months (4 Feb 2024, date last accessed).

[28] Kampmann B , Madhi SA, Munjal I, et al; MATISSE Study Group. Bivalent prefusion F vaccine in pregnancy to prevent RSV illness in infants. N Engl J Med 2023;388:1451–64. 37018474 10.1056/NEJMoa2216480

[29] Simões EAF , Center KJ, Tita ATN, et al Prefusion F protein-based respiratory syncytial virus immunization in pregnancy. N Engl J Med 2022;386:1615–26. 35476650 10.1056/NEJMoa2106062

[30] RSV Prevention. Centre for Disease Control and Prevention. https://www.cdc.gov/rsv/about/prevention.html (4 Feb 2024, date last accessed).

[31] Obando-Pacheco P , Justicia-Grande AJ, Rivero-Calle I, et al Respiratory syncytial virus seasonality: a global overview. J Infect Dis 2018;217:1356–64. 29390105 10.1093/infdis/jiy056

[32] Garg I , Shekhar R, Sheikh AB, et al Impact of COVID-19 on the changing patterns of respiratory syncytial virus infections. Infect Dis Rep 2022;14:558–68. 35893478 10.3390/idr14040059 PMC9394296

[33] Koltai M , Moyes J, Nyawanda B, et al Estimating the cost-effectiveness of maternal vaccination and monoclonal antibodies for respiratory syncytial virus in Kenya and South Africa. BMC Med 2023;21:120. 37004062 10.1186/s12916-023-02806-w PMC10064962

