# Supplementary material for: Respiratory syncytial virus-associated pneumonia in primary care in Malawi
Source: J Trop Pediatr. 2024 Jul 18;70(4):fmae013. doi: 10.1093/tropej/fmae013 (PMC11257717; doi:10.1093/tropej/fmae013)
Supplement: fmae013_Supplementary_Data [file fmae013_supplementary_data.docx]

Supplemental file

Contents

Table of Contents

[S1 Background Health Data 1](#_Toc158930942)

[S1: Immunizations 1](#_Toc158930943)

[S2 Eligibility criteria and exclusion criteria 2](#_Toc158930944)

[S3 Data collection procedure 2](#_Toc158930945)

[Table S1: Microbiologic analyses 3](#_Toc158930946)

[S4: Diagnostic criteria for bacterial and viral pneumonia 6](#_Toc158930947)

[S5 Immunisation status 6](#_Toc158930948)

[S6 Table on monoclonal antibodies and immunisations 7](#_Toc158930949)

## S1 Background Health Data

UNICEF data from 2013 reports an under five mortality rate of 68 per 1,000 live births and under one mortality rate of 44 per 1,000 live births in Malawi. Known factors for childhood pneumonia such as low birth weight (13.5% of all births) malnutrition (13.8% underweight, 47.8% stunting, 4.1% wasting)) and HIV infection (170,000 chidlren living with HIV) are common. Immunisation coverage is high with >90% coverage of BCG and DTP1 and >85% coverage of DTP2and 3, polio, PCV1 Hep B Hib and PCV (PCV was introduced 2011). Rotavirus vaccine coverage was 81%. Immunisation schedule in supplemental file.

HIV prevalence in antenatal clinics in the Mzimba district (where Mzuzu is located) among those who accepted testing was 9.1%[1]. A study in 2011 in Malawi showed that of 5,068 samples from infants <3 months of age , 764 were ELISA positive indicating 15.1% (14.1–16.1%) of mothers were HIV-infected and passed antibodies to their infant. Sixty-five of the ELISA-positive samples tested positive by DNA PCR, indicating a vertical transmission rate of 8.5% (6.6–10.7%). Survey data indicates 64.8% of HIV-infected mothers and 46.9% of HIV-exposed infants received some form of antiretroviral prophylaxis[2]. 

## S1: Immunizations

According to the guidelines developed by the World Health Organization, children are considered to have received all basic vaccinations when they have received a vaccination against tuberculosis (BCG), three doses each of the DPT-HepB-Hib (also called pentavalent) and polio vaccines, and a vaccination against measles. The BCG vaccine is usually given at birth or at first clinical contact, while the DPT-HepB-Hib and polio vaccines are given at approximately age 6, 10, and 14 weeks. The Malawi immunisation programme considers a child to be fully vaccinated if the child has received all basic vaccinations, three doses of the PCV vaccine (also given at age 6, 10, and 14 weeks), and two doses of the rotavirus vaccine (given at age 6 and 10 weeks).

Universal immunisation of children against common vaccine-preventable diseases, namely tuberculosis, diphtheria, whooping cough (pertussis), tetanus, polio, and measles, hepatitis B and Haemophilus influenzae type b (Hib) is given in Malawi. The government of Malawi introduced the pneumococcal conjugate vaccine (PCV 13) and monovalent human rotavirus vaccine (RV1) into the national’s infant immunisation programme in November 2011 and October 2012, respectively. Recent data demonstrates that 96 percent of children received the first dose of the pneumococcal vaccine, 93 percent of children receiving the recommended three doses of DPT-HepB-Hib, and 89 percent the three doses of the pneumococcal vaccine[3]

## S2 Eligibility criteria and exclusion criteria

Children presenting to the primary care facilities were assessed for study enrolment. Children aged 2-59 months were deemed eligible to participate if their main presenting complaints aligned with the current World Health Organization (WHO) clinical case definition of pneumonia; cough or difficulty breathing associated with tachypnoea (>50 breaths per minute in patients aged 2-11 months, or >40 breaths per minute in patients aged 12-59 months) or chest in-drawing (World Health Organization, 2014). Exclusion criteria included those discharged from hospital in the preceding 30 days; those who had completed a course of antibiotics within 14 days of presentation; or those who had received antibiotics prior to clinical assessment for this illness. The study details were discussed with a parent/guardian of any patient who fit the inclusion criteria, and parents provided a written informed consent..

## S3 Data collection procedure

Investigators used a standardised report form to collect demographic data, risk factors and clinical details. Notable factors gathered from standardised report forms include age at presentation, month of presentation, birth maturity and weight. Environmental factors of particular interest included cigarette smoke exposure, household crowding, feeding method, maternal education, oral supplement use and household primary fuel source. All participants had blood samples taken and a malaria rapid diagnostic test. Human immunodeficiency virus (HIV) rapid diagnostic testing was offered to all children over 12 months of age, and HIV polymerase chain reaction (PCR) testing to all children 2-11 months of age. Naso/oropharyngeal swabs were collected for microbiological investigation on-site and for further serological and molecular analysis in Ireland. Blood cultures were processed locally. Blood samples for PCR for Streptococcus pnuemoniae, Staphylococcus aureus and H. influenzae, along with naso/oropharyngeal swabs for respiratory viruses and atypical bacteria were processed in Ireland.

## **Table S1: Microbiologic analyses**

| **Bacterial^[[1]](#footnote-1)^** |  | **Total Population**  **N=488** | | **Not hospitalised (n=432)** | **Hospitalised**  **(n=56)** |  |
| --- | --- | --- | --- | --- | --- | --- |
| S. pneumoniae | n (%) | 4/494 (0.8%) | | 4/438 (0.9%) | 0/56 (0 %) | NS |
| S. auerus | n (%) | 2/494 (0.4%) | | 2/438 (0.5%) | 0/56 (0%) | NS |
| Haemophilus spp. | n (%) | 2/494 (0.4%) | | 2/438 (0.5%) | 0/56 (0%) | NS |
| M. pneumoniae | n (%) | 3/494 (0.6%) | | 3/438 (0.7%) | 0/56 (0%) | NS |
| C._pneumoniae | n (%) | 2/494 (0.4%) | | 2/438 (0.5%) | 0/56 (0%) | NS |
| Any bacterial infection | n (%) | 13/494 (2.6%) | | 13/438 (3.0%) | 0/56 (0%) | NS |
| **Viral^[[2]](#footnote-2)^** |  |  | |  |  |  |
| Adenovirus | n (%) | 82/494 (16.6%) | | 71/438 (16.2%) | 11/56 (19.6%) | NS |
| Bocavirus | n (%) | 100/494 (20.2%) | | 92/438 (21%) | 8/56 (20.5%) | NS |
| CoV* HKU | n (%) | 0/494 (0%) | | 0/438 (0%) | 0/56 (0%) | NS |
| CoV* NL63 | n (%) | 1/494 (0.2%) | | 1/438 (0.2%) | 0/56 (0%) | NS |
| CoV* 229E | n (%) | 1/494 (0.2%) | | 1/438 (0.2%) | 0/56 (0%) | NS |
| CoV* OC43 | n (%) | 1/494 (0.2%) | | 1/438 (0.2%) | 0/56 (0%) | NS |
| Influenza A | n (%) | 76/494 (15.4%) | | 74/438 (16.9%) | 2/56 (15.6%) | NS |
| Influenza AH1 | n (%) | 64/494 (13%) | | 63/438(14.4%) | 1/56 (1.8%) | 0.001 |
| Influenza AH3 | n (%) | 6/494 (1.2%) | | 6/438 (1.4%) | 0/56 (0%) | NS |
| Influenza B | n (%) | 9/494 (1.8%) | | 8/438 (1.8%) | 1/56 (1.8%) | NS |
| HMPV^+^ | n (%) | 73/494 (14.7%) | | 63/438 (14.4%) | 10/56 (15.0%) | NS |
| Parainfluenza 1 | n (%) | 11/494 (2.2%) | | 10/438 (2.3%) | 1/56 (1.8%) | NS |
| Parainfluenza 2 | n (%) | 10/494 (2.0%) | | 10/438 (2.3%) | 0/56 (0%) | NS |
| Parainfluenza 3 | n (%) | 4/494 (0.8%) | | 3/438 (0.7%) | 1/56 (1.8%) | NS |
| Parainfluenza 4 | n (%) | 17/494 (3.4%) | | 15/438 (3.4%) | 2/56 (3.6%) | NS |
| RSV^//^ A | n (%) | 164/494 (33.2%) | | 144/438 (32.9%) | 20/56 (35.7%) | NS |
| RSV^//^ B | n (%) | 50/488 (10.1%) | | 44/438(10%) | 6/56 (10.7%) | NS |
| Rhino/enterovirus | n (%) | 295/494 (59.7%) | | 263/438 (60%) | 32/56 (57.1%) | NS |
| Any virus | n (%) | | 940/488448/494 (93.1%) | 409/432 (94.7%) | 51/56 (91.1%) | NS |

* = Coronavirus

+ = Human metapneumovirus

// = Respiratory syncytial virus

## S4: Diagnostic criteria for bacterial and viral pneumonia

| **Group** | **Criteria** |
| --- | --- |
| **Bacterial pneumonia** | Positive blood culture for significant pathogen (e.g. S. pneumoniae, S.aureus, H. influenzae)  Or  H.influenzae/ S.aureus/ S. pneumoniae detected by blood PCR  With or without  virus detected by RT-PCR |
| **Viral pneumonia** | Negative blood culture for significant pathogen (e.g. S. pneumoniae, S.aureus, H. influenzae,)  AND  H.influenzae/ S.aureus / S. pneumoniae not detected by blood PCR  With  Virus detected by RT-PCR |
| **Unknown etiology** | Negative blood culture for significant pathogen (e.g. S. pneumoniae, S.aureus, H. influenzae)  AND  H.influenzae/ S.aureus/ S. pneumoniae not detected by blood PCR  Without  Virus detected by RT-PCR |

**Table S2 Diagnostic criteria for bacterial and viral pneumonia**

## S5 Immunisation status

| **Variable** | **Statistic** | **Result** |
| --- | --- | --- |
| BCG (>6m only) | n (%) | 317/317 (100%) |
| BCG (>24m only) | n (%) | 109/109 (100%) |
| Pentavalent vaccine – three doses (>6m only) | n (%) | 309/315 (98%) |
| Pentavalent vaccine – three doses (>24m only) | n (%) | 107/109 (98.1%) |
| Polio (>6m only) | n (%) | 315/315 (100%) |
| Polio (>24m only) | n (%) | 109/109 (100%) |
| Pneumococcal (>6m only) | n (%) | 307/315 (97.4%) |
| Pneumococcal (>24m only) | n (%) | 107/109 (98.2%) |
| HiB (>6m only) | n (%) | 290/315 (92%) |
| HiB (>24m only) | n (%) | 102/109 (93.6%) |
| Fully immunised as per Malawi guidelines (>12m only) | n (%) | 216/239 (90.4%) |
| Fully immunised as per Malawi guidelines (>24m only) | n (%) | 98/109 (89.9%) |

**Table S3 Immunisation status**

## S6 Table on monoclonal antibodies and immunisations

|  |  | Doses | Indication |  |
| --- | --- | --- | --- | --- |
| Maternal immunisation | RSVpreF & RSVPreF3 vaccine (Abrysvo) | Single dose | Women who are 32-36weeks pregnant during RSV season | - Phase 3 trials complete and no safety concerns recognized |
| Monoclonal antibodies | Palivizumab (Synagis) | Monthly doses during RSV season | Infants <24 months | - Only approved for infants with conditions that place them at increased risk for severe RSV disease |
|  | Nirsevimab (Beyfortus) & MK-1654 | Single dose is protective for 5 months | Infants >8months born during or entering 1^st^ RSV season | - Higher potency & protect infant during the entire RSV season |
| Immunization of infants with vaccines | Arexvy 2 vaccine |  |  | - Ineffective in infants <6months |

1. Two other bacterial species were identified in the patients who were not hospitalized, but were not clinically associated with pneumonia (E. Coli, and Shigella). [↑](#footnote-ref-1)
2. In 6 cases it was not possible to obtain a valid PCR run [↑](#footnote-ref-2)
